# Small Heat Shock Proteins in Retinal Diseases

**DOI:** 10.3389/fmolb.2022.860375

**Published:** 2022-04-11

**Authors:** Vivian Rajeswaren, Jeffrey O. Wong, Dana Yabroudi, Rooban B. Nahomi, Johanna Rankenberg, Mi-Hyun Nam, Ram H. Nagaraj

**Affiliations:** ^1^ Department of Ophthalmology, Sue Anschutz-Rodgers Eye Center, School of Medicine, Aurora, CO, United States; ^2^ Department of Pharmaceutical Sciences, Skaggs School of Pharmacy and Pharmaceutical Sciences, University of Colorado, Aurora, CO, United States

**Keywords:** small heat shock proteins, glaucoma, diabetic retinopathy, age-related macular degeneration, retina

## Abstract

This review summarizes the latest findings on small heat shock proteins (sHsps) in three major retinal diseases: glaucoma, diabetic retinopathy, and age-related macular degeneration. A general description of the structure and major cellular functions of sHsps is provided in the introductory remarks. Their role in specific retinal diseases, highlighting their regulation, role in pathogenesis, and possible use as therapeutics, is discussed.

## Introduction

The small heat shock proteins (sHsps) family comprises 11 members, namely, HspB1 (Hsp27), HspB2 (myotonic dystrophy kinase-binding protein), HspB3 (Hsp17), HspB4 (αA-crystallin), HspB5 (αB-crystallin), HspB6 (Hsp20), HspB7 (cardiovascular heat shock protein), HspB8 (Hsp22), HspB9 (cancer/testis antigen 51), HspB10 (outer dense fiber protein 1) and HspB11 (Hsp16.2) (Kappé et al., 2003; Schmidt et al., 2016; Zhu and Reiser, 2018). sHspB1, B2 (at low levels), B4, B5, B6, B8 and B11 have been identified in the mammalian retina (O’Reilly et al., 2010; Reddy et al., 2013; Schmidt et al., 2016). sHsps are ATP-independent molecular chaperones (Zhu and Reiser, 2018). sHsps are categorized into two classes (Taylor and Benjamin, 2005). Class I sHsps (HspB1, B5, B6, and B8) are widely distributed in various tissues. However, Class II sHsps (HspB2, 3, 4, 7, 9, and 10) exhibit tissue-specific expression. The molecular masses of the sHsp subunits range between 12 and 43 kDa HspB1, B4, B5, and B11 can form large oligomeric species capable of subunit exchange, which is accelerated under various stress conditions. sHsps have a highly conserved, 80–100 amino acid α-crystallin core domain (ACD), with the notable exception of HspB11 containing the least conserved ACD-like region (Kappé et al., 2010; Bartelt-Kirbach and Golenhofen, 2014). This ACD is flanked by a less conserved N-terminal domain and a C-terminal extension (CTE) (Kriehuber et al., 2010). The CTEs of several sHsps contain an IXI/V motif that contains hydrophobic residues, typically isoleucine or valine, separated by a single residue (Baughman et al., 2020).

sHsps interact with partially unfolded proteins through their surface-exposed hydrophobic residues and prevent protein aggregation (Sharma et al., 2000; Jaya et al., 2009). This property is essential in preventing neurodegenerative and neuromuscular diseases caused by protein aggregation (Carra et al., 2013). The anti-aggregation activity of sHsps can be determined *in vitro* by their ability to inhibit thermal- or chemical-mediated aggregation of client proteins. A comparative analysis has shown that HspB1, B4 and B5 are most effective, B2 and B3 are intermediately effective, and B6, B7, and B8 are moderately effective in their chaperone-like function (Mymrikov et al., 2017).

HspB1, B3, B4 and B5 bind to many heat-sensitive proteins in cells when subjected to thermal stress (Mymrikov et al., 2017). Bcl-2-associated anthanogene (BAG3) is a linker protein of sHsps with large heat shock proteins (Boelens, 2020). BAG3 can interact with HspB2, B5, B6 and B8 and facilitate the removal of misfolded proteins through autophagy (Rusmini et al., 2017; Boelens, 2020). Apart from this, sHsps can also interact with cellular proteins under normal conditions. HspB1, B4, B5 and B6 interact with cytoskeletal proteins such as actin, tubulin, vimentin, nestin, and desmin to maintain the integrity of the cytoskeletal architecture (Mymrikov et al., 2011; Wettstein et al., 2012). In addition, sHsps play an essential regulatory role in maintaining protein homeostasis; by interacting with early misfolded proteins and facilitating their refolding or degradation (mediated by chaperones and co-factors), thereby preventing cell damage (Westerheide and Morimoto, 2005; Webster et al., 2019).

sHsps are also regulators of apoptosis (Takayama et al., 2003; Nagaraj et al., 2016). Several studies have shown that sHsps bind to procaspase-3 and Bax and block apoptosis in cells under various stress conditions (Pasupuleti et al., 2010; Nahomi et al., 2013a; Bakthisaran et al., 2015). sHsps also block TRAIL-, TNF-α- and Fas-induced apoptosis across multiple cell types (Mehlen et al., 1995; Kamradt et al., 2005). Furthermore, sHsps can inhibit apoptosis by activating the cell survival pathway mediated by PI3-K/Akt/PDK1 (Takayama et al., 2003; Pasupuleti et al., 2010).

Previous studies have shown that sHsps are anti-inflammatory proteins. Overexpression of HspB1 ameliorates neuroinflammation caused by ethanol-induced brain injury in mice (Dukay et al., 2021). HspB5 reduces inflammation by decreasing the secretion of proinflammatory cytokines in animal models of multiple sclerosis, ischemia, or nerve crush injury (Kurnellas et al., 2012; Rothbard et al., 2012; Lim et al., 2021). Furthermore, HspB5 protects intestinal mucosa from inflammation in an experimental model in mice (Xu et al., 2019). It also shows anti-inflammatory and otoprotective effects in experimental pneumococcal meningitis (Erni et al., 2019), modulation of the inflammatory response in a mouse model of acute spinal cord injury (Klopstein et al., 2012), and reduction of paralytic symptoms in a mouse model of experimental autoimmune encephalomyelitis through modulation of inflammatory cytokines (Rothbard et al., 2012). Administration of HspB4 showed a reduction in Th1 cytokines in the retina of a mouse model of experimental autoimmune uveitis (Rao et al., 2012).

Apart from the above functions, sHsps are also involved in other cellular processes. HspB1, B5, and B6 have been shown to regulate angiogenesis (Dimberg et al., 2008; Lee et al., 2012; Zhang et al., 2012; Tao et al., 2019), associated with tumor progression and neovascularization in retinal diseases. Several reports are available about the promotion of epithelial to mesenchymal transition in various cells/tissues by HspB1 and B5, which is a phenomenon that supports malignant cell metastasis (Shiota et al., 2013; Cordonnier et al., 2015; Shi et al., 2017a; Li et al., 2017; Chen et al., 2018a; Han et al., 2018; Maksimiuk et al., 2020).

sHsps undergo phosphorylation and other posttranslational modifications in cells. Phosphorylation is mediated through protein kinase A, p38 MAP and MAPKAP kinases (Bakthisaran et al., 2016; Larsen et al., 1997; Edwards et al., 2012), also reviewed in (Bakthisaran et al., 2015). Phosphorylation produces a negative charge, inducing a shift from the oligomeric state to smaller species, as observed in HspB1 and HspB5. The phosphorylation sites identified in sHsps are as follows: HspB1 (serine 15, 78, and 82), HspB4 (serine 45, 122, and serine/threonine 148), HspB5 (serine 19, 45, and 59), HspB6 (serine 16), and HspB8 (serine 24 and threonine 87) (Bakthisaran et al., 2016; Larsen et al., 1997; Shemetov et al., 2011; Edwards et al., 2012; Boelens, 2020; Nath et al., 2021). Phosphorylation of sHsps affects their oligomeric structure and chaperone-like activity. Phosphorylation has been considered a gain-of-function modification in sHsps (Koteiche and McHaourab, 2003; Ecroyd et al., 2007; Ahmad et al., 2008; Jovcevski et al., 2015; Freilich et al., 2018), but there are studies showing conflicting results (Rogalla et al., 1999; Ito et al., 2001; Lelj-Garolla and Mauk, 2006; Shemetov et al., 2008; Mellier et al., 2013). For example, while some studies showed HspB1 phosphomimics (serine residues mutated to aspartic acid) promote the dissociation of oligomers and enhance its chaperone-like activity (Jovcevski et al., 2015; Freilich et al., 2018), other studies showed the opposite effect on the chaperone-like activity (Rogalla et al., 1999; Lelj-Garolla and Mauk, 2006; Mellier et al., 2013). In one study, HspB5 phosphomimics showed more efficient chaperone-like activity against an amyloid-forming protein but reduced activity against another (Ecroyd et al., 2007), suggesting client-specific effects. In another study, a phosphorylation mimic of HspB5 showed a reduced oligomeric size and decreased chaperone-like activity (Ito et al., 2001).

In addition to phosphorylation, other modifications affect the functions of sHsps. HspB1 has been shown to undergo sumoylation, which affects its influence on cell proliferation (Ge et al., 2017). Another modification is glycation. Glycation is a major modification of the human lens proteins HspB4 and HspB5 (Swamy et al., 1992; Cherian and Abraham, 1995), and this modification can either improve or reduce the function of these proteins, depending on which carbonyl compound initiates the glycation (Nagaraj et al., 2012a). Various acyl modifications, such as acetylation, succinylation, malonylation, and propionylation, have also been reported in the human lenses proteins HspB4 and HspB5 (Nagaraj et al., 2012b; Nahomi et al., 2013b; Nandi et al., 2019a; Nahomi et al., 2020). These modifications (acetylation and succinylation) have also been shown to improve the chaperone-like activity of HspB5 (Nahomi et al., 2013a; Nahomi et al., 2013b; Nandi et al., 2019a; Nandi et al., 2019b).

Together, these observations suggest that sHsps are important proteins involved in maintaining cellular homeostasis and that they undergo posttranslational modifications that affect their cellular functions. In the following section, we will review the role of sHsps in three major retinal diseases.

### sHsps in Glaucoma

Immunostaining experiments have demonstrated an upregulation of HspB1 in retinal ganglion cells (RGCs), retinal vessels, and the optic nerve head of primary open-angle glaucoma (POAG) and normal-tension glaucoma patients (Tezel et al., 2000). Studies on nonhuman primate models of glaucoma have shown elevated levels of HspB1 in RGCs and optic nerve fibers (Sakai et al., 2003). Rats with elevated intraocular pressure (IOP) showed increased expression of HspB1 (Huang et al., 2007; Kalesnykas et al., 2007; Windisch et al., 2009; O’Reilly et al., 2010; Chidlow et al., 2014) and phosphorylated HspB1 (Huang et al., 2007) in the nerve fiber layer (NFL), RGCs, and glial cells. In some studies, dynamic expression of HspB4 and B5 was observed in a rat glaucoma model, with initial downregulation followed by upregulation (Steele et al., 2006; Miyara et al., 2008; Guo et al., 2010; Piri et al., 2013; Thanos et al., 2014; Park et al., 2019). Ischemic injury mediated by optic nerve crush or axotomy and steroid-induced ocular hypertension in rats decreased the expression of HspB4 and HspB5 in the RGC layer (Munemasa et al., 2009).

In contrast to HspB1, transcriptional downregulation of HspB5 has been reported in the trabecular meshwork of postmortem glaucomatous human eyes (Comes and Borrás, 2009). Stankowska et al. observed a reduction in HspB5 in human glaucomatous retinas (Stankowska et al., 2019). In rats, episcleral vein cauterization leading to an elevation of IOP over a 7-week period showed a positive correlation between upregulation of HspB4 and HspB5 and decreased loss of RGCs (Anders et al., 2018). The effects of these changes in sHsp levels on the pathogenesis of glaucoma need further studies.

Recent evidence suggests that an immune-mediated response to sHsps contributes to further glaucomatous cell damage. Immunization of Lewis rats with HspB1 caused reductions in RGC number and axonal density (Wax et al., 2008). It has been observed that the titers of autoantibodies against HspB4, B5, and B1 in sera (Tezel et al., 1998; Wax et al., 1998; Wax et al., 2001; Boehm et al., 2012) and against HspB5 in the aqueous humor of normal pressure glaucoma patients are elevated (Joachim et al., 2007). Since serum levels of these antibodies are not correlated with the degree of neuronal cell loss, it is unlikely that an immune response to sHsps arises as a secondary effect following increasing RGC death (Wax et al., 2001). Furthermore, exogenously applied antibodies against HspB4 and HspB5 or HspB1 to human RGC cultures at concentrations similar to those found in glaucoma patients caused apoptotic cell death (Tezel et al., 1998; Tezel and Wax, 2000). In rats, HspB1 injected intraperitoneally led to an upregulation of anti-HspB1 in sera and was correlated with a reduced RGC density after 6 weeks (Joachim et al., 2009). Given that the IOP did not vary in this experiment, the anti-HspB1 antibodies were the likely initiators of the observed RGC loss (Joachim et al., 2009). This is consistent with the finding that inoculation of rat retinal cells *in vivo* with HspB1 antibodies resulted in RGC loss and decreased axon density in the optic nerve 1–4 months later. These results suggested a pathogenic role for sHsp antibodies in glaucomatous cell damage.

There are many reports suggesting that exogenously administered sHsps can be beneficial in preventing cellular damage in glaucoma. Intravenous delivery of HspB5 reduced RGC loss after optic nerve injury at the highest concentration tested and inhibited microglial activation at all concentrations for up to 2 weeks (Wu et al., 2014). Further support for a therapeutic role for HspB5 comes from *in vivo* overexpression of HspB5 in transfected RGCs, which protected against RGC loss for 14 days after injury of optic nerves (Munemasa et al., 2009). Intravitreal injection of HspB5 2 weeks after induction of elevated IOP by episcleral vein cauterization mitigated the loss of rat RGCs and retinal nerve fiber layer thickness (Anders et al., 2017). In ischemic models, intravitreal application of HspB5 improved RGC survival 4 days after acute ocular hypertension (Wu et al., 2012). Intravitreal application of HspB5 immediately after ischemic reperfusion (I/R) injury decreased the RGC loss and retinal thinning 24 h, 1 week, and 1 month later (Yan et al., 2017). Systemic administration of a core peptide of HspB5 in ischemic hypoxic conditions prevented RGC loss in rat retinal explants *ex vivo* and *in vivo.* It inhibited RGC axons and soma degeneration in rodent models of glaucoma (Stankowska et al., 2019). Additionally, the neuroprotective effect of HspB5 extends to other cell types. One study showed that anterior ischemic optic neuropathy induces expression of HspB5 at the optic nerve head (Pangratz-Fuehrer et al., 2011); in this study, intravitreally injected HspB5 for 3 days reduced microglial and astrocyte activation. In addition, intravenous administration of HspB5 every other day for 3 weeks led to the full rescue of oligodendrcytes, which are responsible for the myelination of axons (Pangratz-Fuehrer et al., 2011).

HspB1 is another potential therapeutic target due to its protective role against neurodegeneration. Ischemic retinal preconditioning produced selective upregulation of HspB1 in RGCs and inner retinal layers, which was positively correlated with neuroprotective ischemic tolerance in retinal cells for 72 h (Li et al., 2003). In rats with I/R injury, intravitreal delivery followed by electroporation of HspB1 protected apoptosis of RGCs, supporting the neuroprotective effects of HspB1 (Yokoyama et al., 2001). In optic nerve axotomized rats, induction of HspB1 following intravitreal delivery of simvastatin mitigated RGC apoptosis. The application of quercetin, an inhibitor of HspB1 expression, reversed this effect on RGC survival (Kretz et al., 2006). Other studies showed that optic nerve axotomy induced HspB1 expression in a subset of RGCs; the surviving RGCs showed significantly increased HspB1 expression, indicating a protective effect against apoptosis (Krueger-Naug et al., 2002; Krueger-Naug et al., 2003). Additional evidence for the utility of HspB1 comes from research into axonal regeneration. After transected optic nerves were exposed to peripheral nerve autografts, effective axonogenesis in mature RGCs was strongly correlated with the degree of HspB1 expression (Hebb et al., 2006).

Despite showing beneficial effects in glaucoma, HspB1 has also been claimed to induce the pathogenesis of glaucoma. A previous study showed that intravitreal injection of HspB1 in rats resulted in neuronal and optic nerve damages after 21 days (Grotegut et al., 2020). More recently, it was reported that such damages to the retina occurred via the activation of apoptotic pathways through the activation of NF-κB, and induction of immune response (Grotegut et al., 2021). Furthermore, it is shown that a potential mechanism of HspB1 mediated neurodegeneration is through activation of T cells. A transient 3-week elevation of IOP by microbead injection-induced CD4^+^ T cell migration into the retinal ganglion cell layer (GCL) followed by degeneration of the RGCs and axons up to 8 weeks later. These T cells were shown to be HspB1 specific by cell sorting analysis. Since the retina is immune privileged, one possible explanation for this response is that memory T cells specific to bacterial sHsps gain access to the retina from a compromised retinal blood barrier and are activated by local sHsps. Mice raised without commensal gut microflora did not show these T cell responses or subsequent glaucomatous neural damage, suggesting a role for sensitization of T cells by the gut microbiome in the glaucoma pathogenesis (Chen et al., 2018b). These observations indicate that sHsps play a role in the pathogenesis of glaucoma. The seemingly contrasting effects of HspB1 on RGCs should be investigated further.

### sHsps in Diabetic Retinopathy

HspB4 and HspB5 are expressed in the inner and outer nuclear layers in rodent and human retinas, specifically in RGCs, photoreceptors, astrocytes, and Müller cells (Xi et al., 2003; Fort et al., 2009; Heise and Fort, 2011; Kannan et al., 2016; Ruebsam et al., 2018). Several studies have shown that both HspB4 and HspB5 are upregulated in the retinas of rats with streptozotocin-induced diabetes (Heise et al., 2013). Whether such upregulation has a protective role in the retina has yet to be determined. Murine posterior eye cups exposed to advanced glycation end product-modified proteins showed increased expression of HspB4 but decreased expression of HspB5 (Kase et al., 2011). While retinal HspB4 and HspB5 have neuroprotective roles and support retinal neuronal cell survival, diabetes appears to compromise those effects in rats (Losiewicz and Fort, 2011). This could be because diabetes strongly reduces the chaperone-like function of these sHsps by reducing their solubility and disrupting their interactions with Bax (Losiewicz and Fort, 2011). Another study reported that diabetes compromises the solubility of these proteins in the retina (Reddy et al., 2013), which could have additional negative consequences on their function.

It has been shown that phosphorylation of HspB4 at serine 148 (mice) and threonine 148 (human) is dramatically reduced in diabetic retinopathy, which could increase stress and apoptosis in retinal cells (Ruebsam et al., 2018). HspB4 is highly expressed in Müller glia and protects retinal neurons via a paracrine mechanism (Nath et al., 2022). Further, it has been shown that phosphorylation of threonine 148 in HspB4 enhances its cytoprotective function, which could reduce inflammation in the diabetic retina (Nath et al., 2021). A study using retinas from three different diabetic rat strains showed increased phosphorylation on all three residues (serine 19, 45 and 59) of HspB5 (Heise et al., 2013). In human proliferative diabetic retinopathy, endothelial cells in the epiretinal membranes showed strong immunoreactivity for phosphorylated serine 59 in HspB5 compared to the nondiabetic retina (Dong et al., 2016). Additional studies are required to fully understand the specific role of phosphorylation of HspB4 and HspB5 in the pathogenesis of diabetic retinopathy.

One study reported that intravitreally injected adenovirus encoding HspB4 resulted in reduced vascular leakage and pericyte loss in diabetic mice (Kim et al., 2012). Thus, HspB4 could be essential in inhibiting diabetic retinal pericyte loss and, overall, preventing the pathogenesis of diabetic retinopathy. The levels of HspB1 are decreased in the rat diabetic retina, despite an elevation of its mRNA levels (Reddy et al., 2013), suggesting either enhanced degradation or chemical modification. However, one study in mice (after 10 weeks of streptozotocin-induced diabetes) reported a significant increase in HspB1 mRNA and protein levels in the retina (Pinach et al., 2013). Retinal capillary endothelial cells serve as regulators of vascular wall tension and angiogenesis (Mrugacz et al., 2021), and HspB1 has been shown to play a vital role in maintaining the integrity of these cells (Batulan et al., 2016; Shi et al., 2017b; Rada et al., 2021). However, proinflammatory cytokines that are generally upregulated in the diabetic retina (Joussen et al., 2004; Abu El-Asrar, 2012; Wang et al., 2018) reduce the levels of HspB1 in retinal capillary endothelial cells, which could lead to their apoptosis in diabetic retinopathy (Nahomi et al., 2014). Interestingly, it has also been shown that in glial cells of diabetic rats, HspB1 is upregulated to induce vasoprotective mechanisms to preserve the inner nuclear layer of the retina (Feng et al., 2014). Apart from these sHsps, one study on the diabetic rat retina showed a significant reduction in HspB6, both at the mRNA and protein levels (Reddy et al., 2013), and whether that has any effect on the retina needs to be studied.

Angiogenic growth factors, including vascular endothelial growth factor (VEGF) and platelet-derived growth factor (PDGF), have a role in the proliferative phase of diabetic retinopathy. Angiogenesis can have structural and physiological effects on capillaries and render them dysfunctional. One study found a significant increase in HspB5 in the vitreous fluid of patients with proliferative diabetic retinopathy compared to the nondiabetic control group (Chen et al., 2017). Oxygen-induced retinopathy (OIR) in mice has been widely used as a model for angiogenesis in proliferative diabetic retinopathy. A study on this model reported upregulation of HspB4 and HspB5 (Vähätupa et al., 2018). It has also been observed that under hypoxic conditions, HspB5 is phosphorylated at serine 59 (Ito et al., 1997). Under these conditions, VEGF can stimulate the phosphorylation and functions of HspB5 in capillary endothelial cells (Dimberg et al., 2008). It has been observed that phosphorylated HspB5 can promote VEGF folding and secretion under hypoxic conditions, and in the absence of HspB5, misfolded, monoubiquitinated VEGF may be exported to the cytoplasm, where it is degraded (Kase et al., 2010). Thus, sHsps could play a pivotal role in vascular homoeostasis and retinal angiogenesis in diabetic retinopathy. Furthermore, downregulation of HspB5 in the diabetic retina has been proposed to increase oxidative stress in retinal pigment epithelial cells (Wu et al., 2022). Altogether, the above studies suggest that sHsps play an important role in the pathogenesis of diabetic retinopathy.

### sHsps in Age-Related Macular Degeneration

The pathogenesis of AMD begins with the thickening of Bruch’s membrane due to lipid and protein accumulation forming subretinal deposits, known as drusen. Drusen interferes with fluid efflux from retinal pigment epithelial cells (RPEs) across Bruch’s membrane, leading to increased stress on RPEs. This increased cellular stress leads to proinflammatory signaling combined with an increase in drusen deposition (Bowes Rickman et al., 2013). HspB4 and HspB5 are concentrated in the drusen of macaque monkeys (Umeda et al., 2005), possibly to reduce oxidative damage to RPEs. HspB4 and HspB5 are present in the inner and outer nuclear layers of the retina and RPE (Xi et al., 2003). HspB4 and HspB5 are expressed in the cytosol and mitochondria of RPE cells, and they protect RPEs against oxidative stress and ER stress-induced by ischemic injury and autophagy (Alge et al., 2002).

Since HspB5 is a modulator of angiogenesis by aiding in VEGF folding and secretion (Kase et al., 2010), it might promote choroidal neovascularization in neovascular AMD. In support of this notion is the study by Kase et al. demonstrating that VEGF-A expression is low in HspB5^−/−^ mouse retinas relative to the wild-type, and that laser-induced choroidal neovascularization is reduced in the absence of HspB5 (Kase et al., 2010).

HspB1 has also been detected in the nerve fiber layer, ganglion cell layer and photoreceptors of the retina (Tezel et al., 2000). Oxidative stress in ARPE-19 cells increases HspB1 expression within membrane blebs (Strunnikova et al., 2001). These membrane blebs are inflammatory injury-induced and cause caspase activation, nuclear fragmentation and apoptosis (Wolf and Green, 1999). Animal studies using RPE isolated from mice and human donors and studies applying hydroquinone as an oxidative agent demonstrated that increased oxidative stress on RPE increased the levels of HspB1 phosphorylation and activation (Pons et al., 2010). Another study showed that hydroquinone-induced nonlethal oxidative injury led to *HSPB1* gene transcription and dimer formation and phosphorylation of HspB1 in ARPE-19 cells (Pons et al., 2010). Pons et al. also found that p38 and extracellular signal-regulated kinase (ERK) mediated hydroquinone-induced HspB1 phosphorylation and actin aggregation, revealing ERK to be an upstream regulator of HspB1 (Pons et al., 2010). In addition, it has been shown that HspB5 plays a vital role in subretinal fibrosis associated with neovascular AMD (Ishikawa et al., 2016).

Mini chaperone peptides derived from HspB5 prevent oxidant-induced cell death of RPEs and have therapeutic potential through the binding of proinflammatory mediators (Kannan et al., 2016). The study by Kannan et al. used polycaprolactone nanoparticles loaded with a mini chaperone peptide from either the HspB4 or B5 core domain and demonstrated that this protected primary RPE cells from oxidative stress and was approximately 4-fold more effective than nonencapsulated HspB5 mini chaperone peptide at the same doses (Kannan et al., 2016). Recently, Kannan’s laboratory observed that oxidative stress induces senescence in RPE cells, and a mini chaperone peptide of HspB5 inhibits such senescence (Sreekumar et al., 2022). In addition, Wang et al. demonstrated that recombination of HspB5 with a protein polymer fused with soluble peptide S96 and deblock copolymer SI protected RPE cells from apoptosis induced by chemical stress from H_2_O_2_ (Wang et al., 2014) and its intravitreal injection attenuated NaIO3-induced retinal degeneration in mice (Sreekumar et al., 2018). Another study demonstrated that retinal damage was reduced after administration an HspB5 peptide in a NaIO3-induced retinal damage/degeneration model, and ARPE-19 cells (Raju et al., 2016). Together, these observations suggest that sHsps play a protective role in AMD. Further work is required to fully understand at the molecular level how sHsps help maintain homeostasis in the retina and RPE cells and how alterations in their levels affect the pathogenesis of AMD.

## Concluding Remarks

**FIGURE 1:**
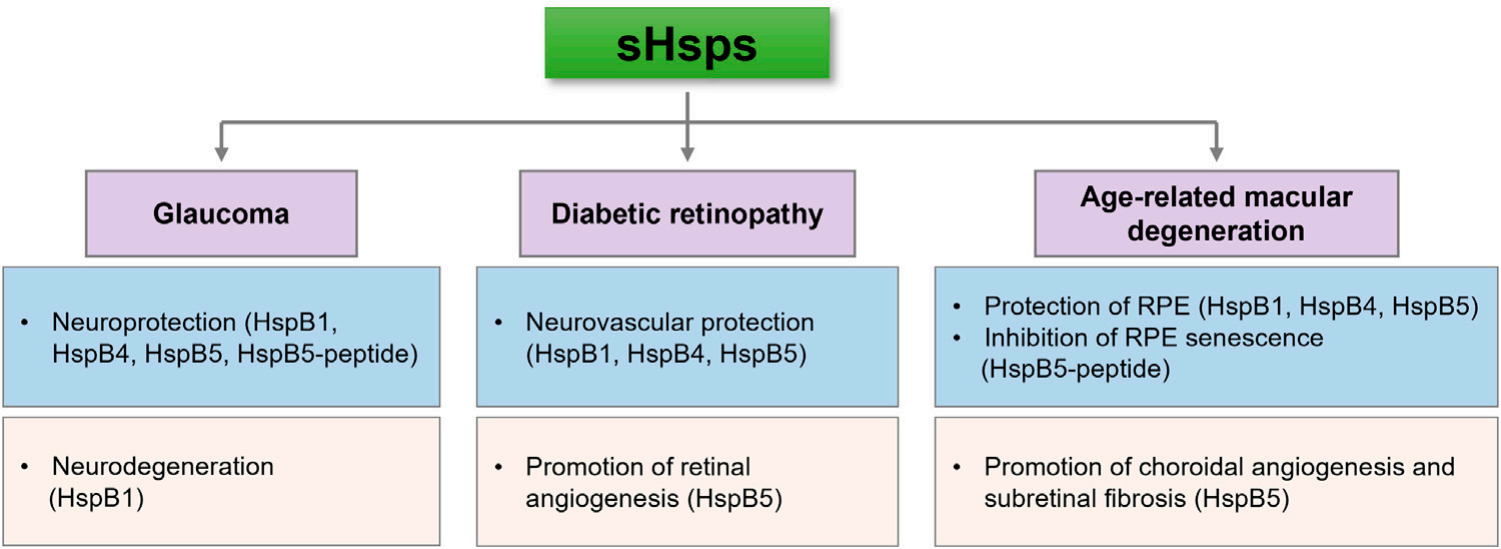
sHsps can inhibit or contribute to the pathogenesis of retinal diseases. This seemingly contradictory ability might depend on their cell type expression, response to stress and posttranslational modifications.

The accumulated evidence confirms that sHsps exhibit molecular chaperone-like functions by interacting with partially unfolded proteins, thereby preventing protein aggregation under stress conditions. In addition, sHsps interfere with apoptosis under various stress conditions at several stages in the intrinsic and extrinsic apoptotic pathways. These properties appear to be important in preventing neurodegeneration and vascular abnormalities in the retina. Moreover, the retina is prone to produce high levels of photooxidative radicals due to its high metabolic activity and exposure to light. Therefore, sHsps might be crucial in protecting retinal cells and RPEs from oxidative damage. sHsps might be useful as therapeutics in retinal diseases due to their neurovascular protective abilities. In addition, recent studies have shown beneficial effects of intraocularly delivered sHsps/sHsp peptides in glaucoma and neovascular AMD. However, sHsps could be pathogenic as well. HspB1 can promote VEGF folding and secretion and thus could promote angiogenesis in proliferative diabetic retinopathy and neovascular AMD. Moreover, HspB1 has been shown to promote RGC death in animal models of glaucoma (Figure 1). Therefore, a better understanding of the molecular mechanisms of sHsp functions in specific cell types of the retina and the impact of disease conditions on their functions is necessary to appreciate their role in retinal diseases fully.
